# Structural, molecular, and functional insights into Schlafen proteins

**DOI:** 10.1038/s12276-022-00794-0

**Published:** 2022-06-29

**Authors:** Ukhyun Jo, Yves Pommier

**Affiliations:** Developmental Therapeutics Branch and Laboratory of Molecular Pharmacology, Center for Cancer Research, NCI, NIH, Bethesda, MD 20892-4264 USA

**Keywords:** Cancer, Cancer therapy

## Abstract

*Schlafen* (*SLFN*) genes belong to a vertebrate gene family encoding proteins with high sequence homology. However, each SLFN is functionally divergent and differentially expressed in various tissues and species, showing a wide range of expression in cancer and normal cells. SLFNs are involved in various cellular and tissue-specific processes, including DNA replication, proliferation, immune and interferon responses, viral infections, and sensitivity to DNA-targeted anticancer agents. The fundamental molecular characteristics of SLFNs and their structures are beginning to be elucidated. Here, we review recent structural insights into the N-terminal, middle and C-terminal domains (N-, M-, and C-domains, respectively) of human SLFNs and discuss the current understanding of their biological roles. We review the distinct molecular activities of SLFN11, SLFN5, and SLFN12 and the relevance of SLFN11 as a predictive biomarker in oncology.

## Schlafen proteins: Understanding structure and function could open therapeutic potential

The diverse roles that Schlafen family proteins play in cell proliferation, immune modulation, and other biological processes make them promising targets for treating and tracking diseases, especially cancer. Ukhyun Jo and Yves Pommier from the National Cancer Institute in Bethesda, USA, review the molecular characteristics and structural features of Schlafen proteins. These proteins take their name from the German word for “sleep”, as the first described Schlafen proteins caused cells to stop dividing, although later reports found that related members of the same protein family serve myriad cellular functions, including in the regulation of DNA replication. A better understanding of Schlafen proteins could open up new avenues in cancer management, for instance, diagnostics that monitor activity levels of one such protein, SLFN11, could help oncologists predict how well patients might respond to anti-cancer therapies.

## Introduction

Repeated genes are classified as tandemly arrayed genes and clustered genes. Tandemly arrayed genes consist of duplications arranged in a head-to-tail fashion. This allows the rapid production of multiple copies of gene products, such as ribosomal RNAs, that act in similar biological functions. In contrast, clustered genes generally express physically and functionally divergent gene products. The genes are functionally linked to each other but play specific roles under different biological circumstances, contributing to genetic complexity and durability^1,2^. *Schlafen* (*SLFN*) genes are clustered genes that are evolutionally conserved in a wide range of vertebrate species^3^ and probably evolved from a common ancestor by multiple unequal recombination events.

**Fig. 1:**
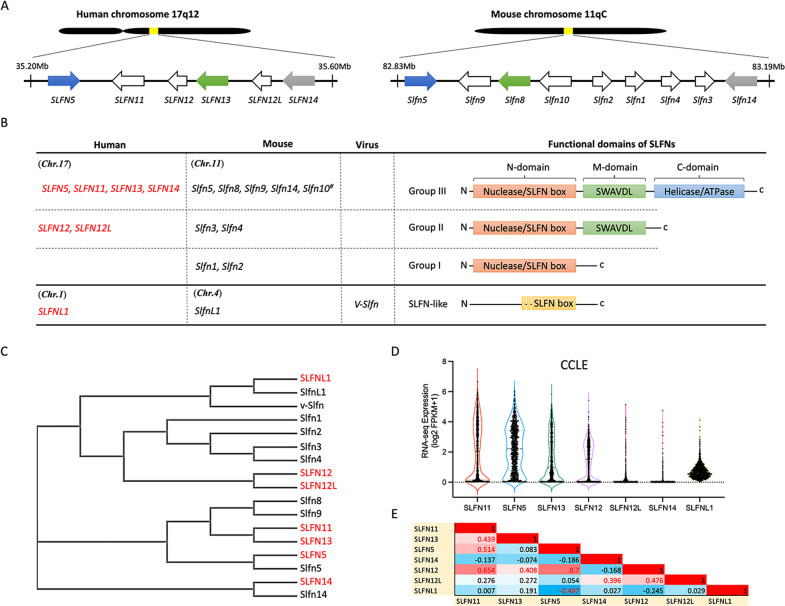
The conserved SLFN family gene cluster. **A** The locations of *SLFN* genes in human and mouse chromosomes. The colors indicate orthologous relationships between human and mouse SLFNs (blank: unknown). **B** Classification of SLFN genes and proteins in human, mouse, and virus. **C** Phylogenetic tree of SLFN proteins generated by ClustalW2. **D** Violin plot of human SLFN mRNA expression in the CCLE cancer cell database (CellMinerCDB, discover.nci.nih.gov/cellminercdb). **E** Correlation of the expression of *SLFN* genes in human.

The first *SLFN* genes (*Slfn1, 2, 3*, and *4*) were identified in mice as a gene family expressed during thymocyte development and immune maturation and preferentially upregulated in the lymphoid cell lineage^4^. Subsequent studies added additional members to the *SLFN* gene family, the members of which were found to exist in gene clusters on the same chromosome in the mouse and human genomes^3,5–8^ (Fig. 1A). Individual *SLFN* genes are differentially involved in multiple cellular processes, including proliferation, differentiation, the immune response, suppression of viral infection, and DNA replication, and are related to chemosensitivity^9–12^. However, the range of functions of SLFNs remains only partially understood.

Here, we describe the main characteristics of the human *SLFN* gene family and focus on the emerging structural insights related to SLFN11’s potential endoribonuclease and helicase/ATPase activity. We also summarize how SLFNs are increasingly being exploited in oncology.

## The *SLFN* family

Over the past two decades, foremost SLFN investigations have focused on the human and mouse *SLFN* genes. Human *SLFNs* (*SLFN5, 11, 12, 12L, 13*, and *14*) are clustered on chromosome 17, and mouse *Slfn* genes (*Slfn1, 2, 3, 4, 5, 8, 9*, and *14*) and the pseudogene (*Slfn10*) are clustered on chromosome 11 (Fig. 1A). In addition, a *SLFN*-like gene containing a partial SLFN box has been identified in the genomes of various species, including human, mouse, and orthopoxvirus (Fig. 1B)^13^.

SLFN proteins are classified into three groups based on their structures and functional domains (Fig. 1B). Group I SLFNs consist of the common N-domain region containing a nuclease structure and a unique SLFN box conserved in all SLFN proteins^2,14^. Group II SLFNs contain the N-domain and a linker middle domain region (M-domain), including a SWAVDL motif and a potential protein-interacting region^14^. Group III SLFNs form the largest subgroup. They include a third functional putative helicase/ATPase C-terminal domain with Walker A/B motifs^15^. SLFN-like proteins contain partially conserved amino acid sequences with the SLFN box, but their biological activity remains unknown.

SLFN proteins are differentially localized in cells, reflecting their putative cellular functions. Mouse Group 1 and II SLFNs are predominantly found in the cytoplasm, while Group III SLFNs are present in the nucleus^7^. In contrast to mouse *Slfn* genes, human *SLFN* genes only encode polypeptides belonging to Group II (SLFN12) and Group III (SLFN5, 11, 13, and 14). While SLFN12 and SLFN13 are cytoplasmic, SLFN11, SLFN14, and SLFN5 are present in the nucleus because of their nuclear localization signal^2,11,14,16,17^. Although SLFN11 is primarily detected in the nucleus by immunostaining and immunofluorescence, it also modulates proteotoxic stress control and protein translation in the cytoplasm^18,19^.

Phylogenic analyses have shown homologous relationships between human, mouse, and virus SLFN protein sequences (Fig. 1C). Human SLFN5 and SLFN14 have direct mouse orthologs (Slfn5 and Slfn14, respectively). Mouse Slfn8 and SLFN13 are also functional homologs^2^. Human SLFN12/12L and SLFN11/13 show sequence similarity with mouse Slfn3/4 and Slfn8/9, respectively. Whether mouse Slfn9 is the ortholog of SLFN11 remains to be determined. Humans do not have gene orthologs or homologs for mouse Slfn1 and Slfn2.

Although *SLFNs* are clustered on the same chromosome, transcription profiling data in the Cancer Cell Line Encyclopedia (CCLE) cancer cell line database^20^ show that each *SLFN* is expressed independently of the others (Fig. 1D, E). *SLFN11*, *SLFN5*, *SLFN13*, and *SLFN12* exhibit a wide range of transcription levels in cancer cells, whereas *SLFN12L*, *SLFN14* and *SLFNL1* are barely expressed in cancer cell lines.

## Structure of SLFN proteins

Structural and biochemical studies have begun to reveal the molecular characteristics of SLFN proteins. The N- and C-domains appear to function as a nuclease and a helicases/ATPase, respectively, while the M-domain may be a linker connecting the two N- and C-terminal enzymatic domains and may potentially interact with other proteins.

### The N-domain of SLFNs: an endoribonuclease domain

**Fig. 2:**
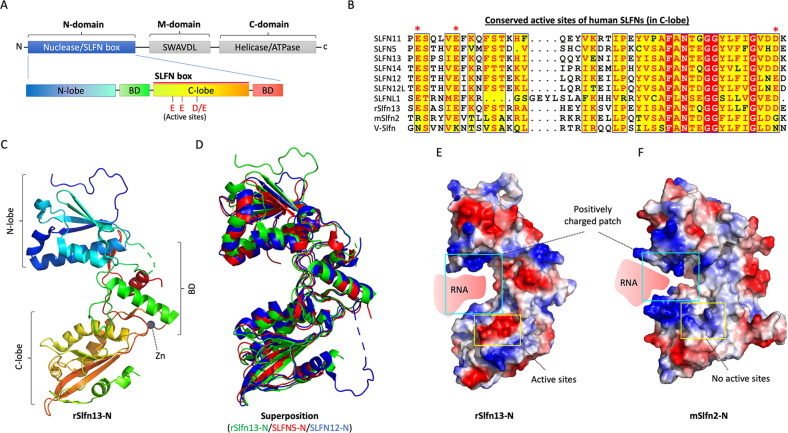
The N-domain of SLFNs. **A** Schematic diagram of an SLFN protein. The expanded diagram shows the N-domain of SLFN proteins with the conserved active site. **B** Sequence alignment of the conserved active site in human, mouse, and virus SLFNs. The sequence alignment was generated using ESPript. **C** Ribbon diagram of the crystal structure of rSlfn13-N (PDB: 5YD0). BD: bridging domain. **D** Superimposition of rSlfn13-N, SLFN5-N (PDB: 6RR9), and SLFN12-N (PDB: 7LRE). **E** The electrostatic surface potentials of rSlfn13-N. The colors indicate electric charge (red: negative and blue: positive). **F** The electrostatic surface potentials of mSlfn2-N (1-378) (modeled by AlphaFold: AF-Q9Z0I6-F1).

SLFN14 purified from rabbit reticulocytes shows novel endoribonuclease activity against rRNA and ribosome-associated mRNAs 21. The N-domain of SLFNs has been revealed as a key domain related to tRNA/rRNA endoribonuclease activity^2,21^. Structural analyses of rat Slfn13 (14~353 residues), which is the homolog of human SLFN13 and mouse Slfn8 (Fig. 2A, C), have shown that the N-domain consists of two lobes (N-lobe and C-lobe) between two bridging domains (BDs) (Fig. 2A). Structural conservation of the SLFN-N domain is also observed in SLFN12 and SLFN5, which shows only slightly different conformations (Fig. 2D)^14,22,23^. Notably, the C-lobe region (also referred to as the SLFN box) with high sequence identity between SLFN proteins includes the conserved active residues (EED: Glu-Glu-Asp) for ribonuclease activity (Fig. 2A, B). The SLFN-N domain has a U-shaped architecture and binds nucleotides via a positively charged patch in the valley (Fig. 2E). In contrast, the electrostatic surface of the ribonuclease active site is negatively charged, and its enzymatic activity relies on Mn^2+/^Mg^2+ ^^2^ (Fig. 2E). Although the active site is conserved among SLFN family members, the enzymatic activity of SLFNs varies. SLFN11 selectively suppresses the cellular type II tRNAs that are utilized to synthesize DNA repair response proteins such as ATR and ATM and HIV proteins^21^. SLFN13 cleaves the acceptor stem of tRNAs, implying that SLFNs recognize the secondary or tertiary structure of substrate RNAs^2^. In in vitro experiments, SLFN12 cleaves rRNA as an active RNase complex with phosphodiesterase 3A (PDE3A), enhancing DNMDP (6-(4-(diethylamino)-3-nitrophenyl)-5-methyl-4,5-dihydropyridazin-3(2H)-one)-induced cancer cell death^14^. Rabbit SLFN14 cleaves rRNA and ribosome-associated mRNA in a manner dependent on Mg2+ and Mn2+ in reticulocytes^24^. However, the substrate specificities of SLFNs have not yet been fully defined. In contrast to the other SLFNs, SLFN5 does not have endoribonuclease activity against tRNAs^2^, although its active site is conserved, implying that it might target single-stranded or double-stranded DNA. Conversely, the active site of mouse Slfn2 is positively charged (Fig. 2F). Yue et al. discovered that mouse Slfn2 shields tRNAs from cleavage by ribonucleases activated by oxidative stress in T cells, thereby counteracting translation inhibitory effects^25^. These observations suggest that the functions of SLFNs have evolutionally adapted according to their environments.

In addition, a putative zinc finger motif has been identified on the backside valley in SLFN5, SLFN12, and rSlfn13 (Fig. 2C)^2,14,23^, implying that the putative zinc finger might help in the recognition of nucleotide targets or assist protein folding and nuclease/ATPase activity in the M and C domains. The zinc finger motif is also conserved in all human and mouse SLFNs, indicating that nucleotide-binding capacity is one of the molecular features of SLFNs.

### The M-domain of SLFNs: a linker and protein-interacting domain

**Fig. 3:**
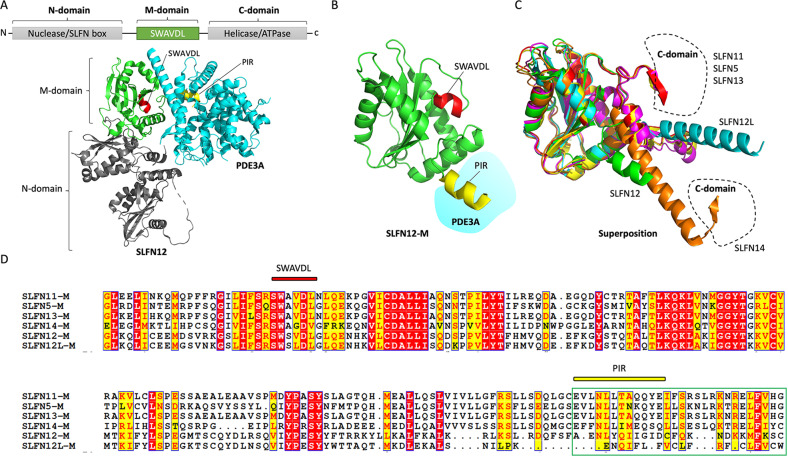
The M-domain of SLFNs. **A** Schematic diagram of the M-domain of SLFN proteins. The ribbon diagram shows the cryo-EM structure of SLFN12-PDE3A (PDB: 7LRD) including the M-domain (green), SWAVDL motif (red), and PIR (PDE3A interacting region; yellow). **B** The expanded M-domain of SLFN12. **C** Superimposition of SLFN12-M (PDB: 7LRE), SLFN11-M (AlphaFold: AF-Q7Z7L1-F1), SLFN5-M (AlphaFold: AF-Q08AF3-F1), SLFN12L-M (AlphaFold: AF-Q6IEE8-F1), SLFN13-M (AF-Q68D06-F1), and SLFN14-M (AlphaFold: AF-P0C7P3-F1). **D** Sequence alignment of the M-domains in human SLFNs. The sequence alignment was generated using ESPript.

Recently, the M-domain of human SLFN12, which binds PDE3A through its PDE3A interacting region (PIR), has been defined using cryo-electron microscopy^14^ (Fig. 3A, B). The M-domain of human SLFN12 also includes the conserved SWAVDL sequence common to all SLFN family members (Fig. 3B, D). The C-terminal region of the M-domain of human Group II SLFNs (SLFN12 and SLFN12L) is structurally distinct from that of the Group III SLFNs (SLFN11, SLFN5, SLFN13, and SLFN14). Group II SLFNs show a stretched-out helix end (Fig. 3C), whereas the M-domains of the Group III SLFNs are kinked where they connect to their putative helicase/ATPase in the C-domain^23^. Among the Group III SLFNs, SLFN14 exhibits a longer helix, with a different connection between its linker region and its C-domain (Fig. 3C).

SLFN14 utilizes its M-domain to bind to ribosomes, and alteration of the M-domain reduces endonucleolytic RNA cleavage activity^24^, indicating that the M-domain of SLFNs might be a docking site for nucleic acids or for functional cofactors, as seen in the SLFN12-PDE3A complex. Molecular interactions between SLFN12 and ribosomal proteins (RPS27A, RPS6, and RPL7A) have also been detected after treatment with 17-β-estradiol (E2), thereby inhibiting the translation of ER-mediated antiapoptotic proteins (Bcl-2 and Mcl-1)^26^. Similarly, SLFN13 might negatively modulate translation by cleaving ribosomal RNAs^2^. Furthermore, SLFN11 suppresses the proliferation of hepatocellular cancer cells by interacting with the ribosomal protein S4 X-linked (RPS4X), resulting in attenuation of S6 and eIF4E phosphorylation in the ribosome complex and inhibition of the mTOR signaling pathway^27^. SLFN11 also functionally associates with protein folding and translation initiation complexes to protect cells from proteotoxic stress^18^. Further studies are warranted to clarify how the M-domain of SLFNs affects ribosome interactions.

### The C-domain of group III SLFNs: a putative helicase/ATPase domain

**Fig. 4:**
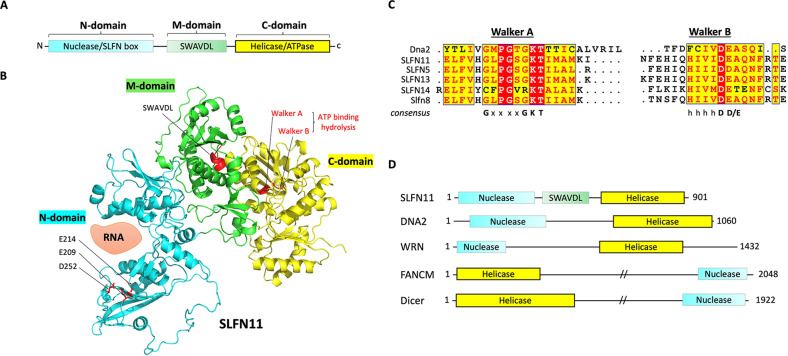
The C-domain of SLFNs. **A** Schematic diagram of the C-domain of SLFN proteins. **B** The ribbon diagram shows the modeled structure of SLFN11 (AlphaFold: AF-Q7Z7L1-F1). The annotations indicate the localization of key elements in SLFN11. **C** Sequence alignment of the Walker A/B motifs in human SLFNs, mouse Slfn8, and mouse Dna2. The sequence alignment was generated using ESPript. **D** Schematic diagram of selected proteins targeted by DNA/RNA helicases.

The C-domain of Group III SLFNs bears homology to superfamily I RNA/DNA helicases^5^ (Fig. 4A). Protein modeling shows that the C-domain region of SLFN11 structurally resembles the structure of Dna2, a nuclease-helicase that controls genomic integrity^28,29^ (Fig. 4B). The C-domain of SLFN11 contains conserved Walker A and B motifs, suggesting ATPase activity. This ATPase motif characterizes all Group III SLFNs (Fig. 4C), implying that the C-domain of SLFNs might function in chromatin remodeling via RNA/DNA helicase activity, as seen in other helicases, including DNA2, WRN, FANCM, and Dicer (Fig. 4D). The ATPase activity of the SLFN helicase motif has been established by using SLFN11 variants (K605M/D668A and E669Q) in the Walker A and B motifs^17,30^. The helicase activity is required for SLFN11-mediated chemosensitivity to DNA-damaging agents^17^ and replication fork degradation. It abolishes the recruitment of RAD51 at stalled forks in Fanconi anemia cells, thereby exposing the stalled forks to the nucleases MRE11 and DNA2^31^.

The ATPase activity of SLFN11 is essential for the killing of cancer cells in response to replicative DNA-damaging agents and chromatin opening, which leads to lethal replication arrest and activation of cellular stress response genes in the FOS-JUN pathways^17^. In addition, the D668A/E669A mutant of SLFN11 fails to attenuate prototype foamy virus (PFV) replication^32^, suggesting that the ATPase activity of the C-domain plays a role in the antiviral properties of Group III SLFNs^19,33,34^.

SLFN5 binds to HSV-1 DNA to interrupt its accessibility to RNA polymerase II, thereby blocking the transcription of viral promoters in host cells^35^. SLFN5 also negatively controls STAT1-mediated transcriptional activation of IFN-stimulated genes and *ZEB1* transcription, suppressing the antitumor immune response in glioblastoma cells and the mesenchymal-epithelial transition^36,37^. The inability of SLFN5 C-domain mutant to inhibit transcription implies the importance of its helicase/ATPase activity. Further studies are warranted to determine how the putative ATPase activity regulates DNA replication and transcription and how the C-domain of Group III SLFNs makes them functionally distinct from the Group I and II SLFNs, as helicases commonly participate in various cellular processes, such as DNA replication, transcription, translation, recombination, DNA repair, and ribosome biogenesis, by remodeling RNA/DNA strands using ATP hydrolysis^38–41^.

### Putative posttranslational modifications of SLFNs

Posttranslational modifications such as phosphorylation, acetylation, and ubiquitination fine-tune the activity and functional localization of helicases according to different steps of the cell cycle and biological processes^41,42^. SLFN11 has been shown to be phosphorylated in both its N-domain (S219 and T230) and its C-domain (S753)^43^. Upon DNA damage, protein phosphatase 1 catalytic subunit γ (PPP1CC) dephosphorylates SLFN11 to increase its activity, thereby sensitizing cancer cells to the topoisomerase I inhibitor, camptothecin. Similarly, SLFN12 dephosphorylation (S368 and S573) is induced by cytotoxic PDE3A modulators, promoting its RNase activity and leading to cell death^44^. Ubiquitination has been observed for SLFN14 when it is misfolded due to missense mutations^45^. Hence, it is likely that posttranslational modifications regulate the cellular function of SLFN proteins.

### v-Slfn and SLFNL1

As mentioned above in the review of the classification of the SLFN family (Figs. 1B and 2B), a partially conserved SLFN sequence has been detected in the C-terminal region of SLFN-like proteins in human, mouse, and virus. The expression of virus Schlafen (v-Slfn) was first observed during infection by the camelpox virus, in which v-Slfn modulated virulence and showed predominantly cytoplasmic localization in the host cells^13^. v-Slfn is conserved across orthopoxviruses. Notably, v-Slfn encodes a 57 kDa protein consisting of poxin fused with the SLFN N- and C-terminal domains^46^. The poxin domain, which includes a viral cyclic guanosine monophosphate-adenosine monophosphate (cGAMP) nuclease, inactivates the cGAS-STING pathway, consistent with the importance of the cGAS-STING signaling pathway for antiviral responses against orthopoxviruses. However, details of the functional roles of the SLFN domain in v-Slfn remain to be elucidated.

Human and mouse also express the SLFN-like protein SLFNL1, which resembles v-Slfn (Figs. 1B and 2B). SLFNL1 contains a partial SLFN domain in the C-terminus and an unknown N-terminal domain, thus being evolutionally divergent from v-Slfn. Further studies are warranted to understand the underlying molecular mechanism of SLFNL1 in cells.

## Exploitation of human SLFNs for cancer therapy

The diverse functions of SLFNs in key cellular processes, such as DNA replication, cell proliferation, transcription, protein folding, and cell motility, highlight the potential of SLFNs as therapeutic targets and biomarkers for diagnosis, therapeutic decision, and prognosis.

### SLFN11

#### Replication checkpoint activity of SLFN11

**Fig. 5:**
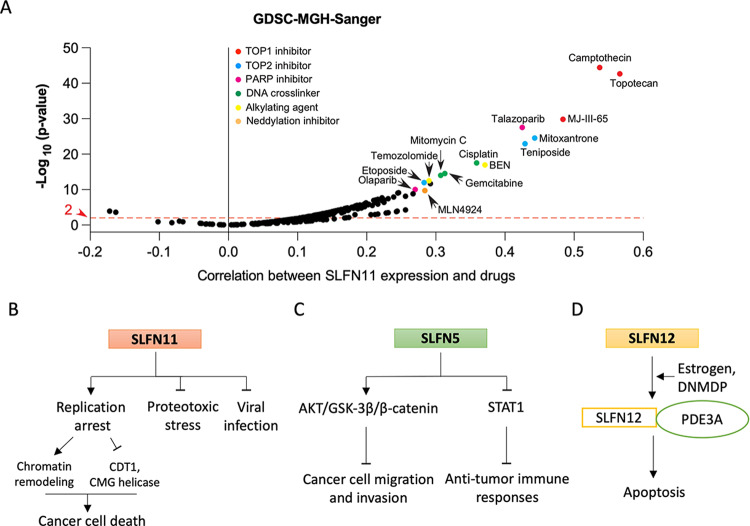
SLFNs in cancers. **A** Correlation of SLFN11 expression with sensitivity to anticancer drugs in the GDSC-MGH-Sanger database (each point is a drug; *n* = 297) analyzed using CellMinerCDB (https://discover.nci.nih.gov/cellminercdb). **B** Proposed signaling model for SLFN11 as a replication checkpoint. **C** Proposed signaling model for SLFN5 as a tumor suppressor and oncogene. **D** Proposed functional model for SLFN12 with PDE3A activated by estrogen and DNMDP.

During the last decade, SLFN11 has been extensively studied due to its relevance in cancer research. SLFN11 acts as a negative replication checkpoint in response to replication stress in parallel to the ATR pathway^47,48^. When replication stress is induced by endogenous or exogenous factors, SLFN11 proteins are recruited and accumulate in the proximity of DNA lesions in coordination with the single-stranded binding protein replication protein A (RPA), forming foci^30,49^. Chromatin-bound SLFN11 destabilizes replication by interacting with RPA, the replicative helicase minichromosome maintenance complex component 3 (MCM3), and chromatin licensing and DNA replication factor 1 (CDT1), leading to irreversible replication arrest^17,50^, whereas ATR activates the downstream kinase CHK1 to transiently arrest the cell cycle and enable repair. SLFN11 simultaneously targets chromatin to modify structural accessibility and activate transcription of immediate early genes (IEGs), including *JUN, FOS, EGR1, NFKB2, ATF3, CDKN1A* (p21^WAF1^), and the growth arrest and DNA damage-inducible gene *GADD45*^17,30,50,51^ (Fig. 5B). In addition, a recent study in Fanconi anemia cells showed that SLFN11 hinders the binding of the single-strand binding recombination protein RAD51 at stalled forks and destabilizes nascent DNA tracts, leading to degradation of the stalled forks by the nucleases MRE11 and DNA2^31^.

#### SLFN11 as a biomarker predicting sensitivity to DNA-damaging agents (DDAs)

Given that SLFN11 is a key responder to replication stress, its expression status is being actively investigated as a biomarker for drug selection and prognosis in cancer therapy with broadly used DNA-damaging agents (DDAs), including topoisomerase I (TOP1) inhibitors (topotecan, irinotecan, and indotecan), TOP2 inhibitors (etoposide, doxorubicin, and epirubicin), alkylating and crosslinking agents (cyclophosphamide, temozolomide, cisplatin, carboplatin, and oxaliplatin), and DNA synthesis inhibitors (5-fluorouracil, gemcitabine, cytarabine, and hydroxyurea)^12,52–54^ (Fig. 5A). SLFN11 expression is also correlated with vulnerability to poly-(ADP)-ribose polymerase (PARP) inhibitors (olaparib, veliparib, talazoparib, and niraparib)^47,54,55^. Conversely, a lack of SLFN11 expression can cause resistance to DDAs^12,52^. *SLFN11* mRNA expression data in the National Cancer Institute Antitumor Cell Line Panel (NCI-60), Cancer Cell Line Encyclopedia (CCLE), and Genomics of Drug Sensitivity in Cancer (GDSC) datasets show that SLFN11 is not expressed in ~50% of cancer cells that exhibit poor response to clinically used DDAs. The relationship between SLFN11 expression and sensitivity to DDAs has been assessed in colorectal, ovarian, lung, breast, head and neck, gastric, esophageal, and prostate cancers, as well as sarcomas^56–66^.

The lack of SLFN11 expression is primarily due to epigenetic changes in DNA methylation in the *SLFN11* gene promoter^67^. The promoter CpG islands of *SLFN11* are frequently hypermethylated in colorectal cancers, leading to poor prognosis and chemoresistance^68,69^. The gene body of SLFN11 is also targeted by the histone modifier EZH2 (enhancer of zeste homology 2) during acquired chemoresistance in small-cell lung cancer (SCLC) cells, which increases H3K27me3 and local chromatin condensation^70^. Since epigenetic changes can be easily detected in patient samples, DNA methylation can be utilized as a surrogate marker to determine susceptibility to DDAs. Therapeutic strategies using inhibitors of EZH2, histone deacetylases (HDACs), and DNA methyltransferases (DNMTs) have been shown to reactivate SLFN11 expression and overcome resistance to DDAs^68,70,71^.

Given the therapeutic relevance of SLFN11 in oncology, the way to determine SLFN11 DNA, RNA, and protein expression in clinical samples are being actively investigated. Advances in microarray and sequencing technologies have made genome-wide profiling to detect *SLFN11* mRNA and gene methylation. Immunohistochemistry (IHC) has also been successfully evaluated as a biomarker in two clinical trials of PARP inhibitors (NCT03880019 and NCT04334941) with various tumor types^72,73^. The reliability of determining SLFN11 expression status by IHC has been confirmed in a large number of patient tumors with various histologies^48,56,62,72,73^. Thus, IHC could readily be applied to evaluate the expression of SLFN11 in the clinical setting.

#### Therapeutic strategies including SLFN11 and ATR inhibitors

The chemoresistance of SLFN11-negative cancer cells can be overcome by combining DDAs with ATR inhibitors^47,48,50,74,75^. Because SLFN11-deficient cancer cells rely on the ATR pathway to modulate their DNA replication and damage repair processes in response to replication stress, the combination of ATR inhibitors with DDAs is selectively active in SLFN11-negative cancer cells^47,76^. These observations suggest that the expression status of SLFN11 can be used alongside cancer therapy with ATR inhibitors, which are in late-phase clinical development.

#### Overexpression of SLFN11

Overexpression of SLFN11 is observed in leukemia and sarcoma cells, implying that it might be involved in the development of certain cancer types^48,53,77,78^. The oncogenic *EWS-FLI* fusion transcriptionally activates *SLFN11* expression in Ewing’s sarcoma^78^. In addition, gain-of-function mutations in the JAK signaling pathway in acute leukemia cells have recently been shown to cause high expression of SLFN11 due to abnormal activation of the upstream ETS transcription factor^77^. How overexpression of SLFN11 is related to tumorigenesis and the cytoplasmic roles of SLFN11 need to be studied in further research.

#### Cytoplasmic roles of SLFN11

In addition to its replication stress checkpoint functions, SLFN11 interacts with ribosomal protein S4 X-linked (RPS4X) and suppresses the mTOR signaling pathway in hepatocellular carcinoma (HCC), inhibiting HCC growth and metastasis^27^. SLFN11 also protects cells from proteotoxic stress caused by the accumulation of unfolded proteins, while its deficiency increases the cellular levels of ubiquitin conjugates due to uncontrolled endoplasmic reticulum stress and protein quality control^18^. A difference in the activity of the proteotoxic stress response pathway was recently found to explain why the clinically developed and first-in-class inhibitor of the ubiquitin-activating enzyme UBA1, TAK-243, selectively targets SLFN11-deficient cells^18^. Further studies are warranted to identify anticancer drugs that function specifically in SLFN11-negative cancers that could be used alone or in combination with TAK-243.

### SLFN5

As a member of SLFN Group III, SLFN5 shares most of the structural domains of SLFN11, SLFN13, and SLFN14. However, the endoribonuclease activity of SLFN5 appears defective, suggesting that SLFN5 is functionally unique in the SLFN family^2,23^. Nevertheless, SLFN5 expression is still induced by interferon (IFN), and IFN-activated SLFN5 localizes mainly in the nucleus and suppresses the anchorage-independent growth of melanoma cancer cells^79^. Negative regulation of cell motility and invasiveness has also been reported for SLFN5 in renal cell carcinoma (RCC), in which SLFN5 expression is positively correlated with survival benefit^80^.

Mechanistically, SLFN5 also interacts with the NOTCH/TGF-β signaling pathway and suppresses matrix metalloproteinases-1 (MMP-1) and MMP-13, which are required for the degradation and rearrangement of extracellular matrix (ECM) proteins, thereby blocking morphological changes. SLFN5 also downregulates MMP14 expression through inhibition of the β-catenin pathway^81^ (Fig. 5C). As a transcriptional repressor, SLFN5 prevents epithelial-mesenchymal transition (EMT) in breast cancer and targets the *ZEB1* promoter to abrogate *ZEB1* transcription and the downstream PTEN/AKT/cyclin D1 signaling cascade, ultimately prompting cancer cell death^37,82^. These observations suggest that SLFN5 could be exploited as a biomarker for cancer therapy with IFN stimulation.

SLFN5 expression has been correlated with cancer progression. In gastric cancer (GC) cells, SLFN5 has been associated with the aggressive transition from intestinal metaplasia to GC^83^. In glioblastoma, SLFN5 promotes tumor formation, growth, and invasion, suppressing STAT1-driven gene transcription^36^ (Fig. 5C). Oncogenic SLFN5 expression has also been observed in castration-resistant prostate cancer patients with poor outcomes. A direct interaction between SLFN5 and ATF4 has been proposed to regulate the L-type AA transporter LAT1, which activates the mTOR signaling pathway^84^. The apparently divergent roles of SLFN5 as a tumor suppressor and tumor promoter need to be investigated in further studies.

### SLFN13 and SLFN14

Yang et al. provided the first structural insights demonstrating that the conserved N-domain of SLFN cleaves tRNA and rRNA by endoribonuclease activity^2^. In glioblastoma, high expression of *SLFN13* mRNA is detected along with poor overall survival^36^. In thrombocytopenia patients, SLFN14 mutations (K218E, K219N, and V220D) localized near the active sites of the SLFN box have been identified^16^. These variants of SLFN14 are associated with platelet secretion defects, suggesting that *SLFN14* mutations might be related to preneoplastic changes.

### SLFN12 and SLFN12L

SLFN12 is a human Group II SLFN lacking the C-terminal helicase domain (Fig. 1A). SLFN12 might play similar roles to the Group III SLFNs SLFN11, 5, 13, and 14 by associating with a functional partner molecule that compensates for the missing C-terminal domain^85^. SLFN12 interacts with phosphodiesterase 3A (PDE3A), which is a regulator of the development of interstitial cells of Cajal and gastrointestinal stromal tumors (GISTs)^85,86^. SLFN12 has been proposed as a therapeutic target and predictive biomarker for PDE3 inhibitors such as zardaverine and quazinone^87^. The drug activity of PDE3 inhibitors has also been shown to be enhanced by coexpression of PDE3A and SLFN12^85,87,88^. Diverse chemical modulators, including 17-β-estradiol (E2), anagrelide, nauclefine, and DNMDP (6-(4-(diethylamino)-3-nitrophenyl)-5-methyl-4,5-dihydropyridazin-3(2H)-one), lead to apoptotic cell death by enhancing the molecular interaction between PDE3A and SLFN12 and increasing SLFN12 RNase activity independent of any inhibition of PDE3A enzymatic activity^14,22,26,85,89,90^ (Fig. 5D). Structural studies revealed that the M-domain of SLFN12 is required for binding to PDE3A^14,22^. SLFN12 expression is also correlated with favorable therapeutic outcomes in lung, prostate, and breast cancers^91–93^.

The expression of SLFN12L, another human Group II SLFN, is associated with the transition of preneoplastic cells to gastric cancer cells during *Helicobacter* infection^94,95^. However, the details of how SLFN12L mechanistically regulates cell transformation remain unclear.

## Conclusions

SLFNs have emerged as biomarkers and therapeutic targets and have been linked with immune responses and suppression of viral infections. Structural studies have provided fundamental molecular clues for how the N- and M-domains of SLFNs can modulate cellular processes through RNA/DNA and functional cofactors to inhibit abnormal cellular replication and viral replication and promote cell death. However, further studies focusing on the C-domain of SLFNs are warranted to further understand the biological roles of SLFNs, and such studies will uncover how SLFNs are structurally and functionally involved. The cellular interactors and posttranslational modifications of SLFNs also remain to be fully established.
